# Students’ intelligence test results after six and sixteen months of irregular schooling due to the COVID-19 pandemic

**DOI:** 10.1371/journal.pone.0281779

**Published:** 2023-03-08

**Authors:** Moritz Breit, Vsevolod Scherrer, Joshua Blickle, Franzis Preckel

**Affiliations:** 1 Department of Psychology, University of Trier, Trier, Germany; 2 Department of Psychology, Chemnitz University of Technology, Chemnitz, Germany; French National Center for Scientific Research (CNRS) & University of Lyon, FRANCE

## Abstract

The COVID-19 pandemic has affected schooling worldwide. In many places, schools closed for weeks or months, only part of the student body could be educated at any one time, or students were taught online. Previous research discloses the relevance of schooling for the development of cognitive abilities. We therefore compared the intelligence test performance of 424 German secondary school students in Grades 7 to 9 (42% female) tested after the first six months of the COVID-19 pandemic (i.e., 2020 sample) to the results of two highly comparable student samples tested in 2002 (*n* = 1506) and 2012 (*n* = 197). The results revealed substantially and significantly lower intelligence test scores in the 2020 sample than in both the 2002 and 2012 samples. We retested the 2020 sample after another full school year of COVID-19-affected schooling in 2021. We found mean-level changes of typical magnitude, with no signs of catching up to previous cohorts or further declines in cognitive performance. Perceived stress during the pandemic did not affect changes in intelligence test results between the two measurements.

## Introduction

The ongoing COVID-19 pandemic and the associated countermeasures have caused many temporary but far-reaching changes in societal processes around the world, affecting work, culture, social life, and education. Like many other institutions, schools were often unprepared for a fundamental change and partial shutdown of their operations, leading to a prolonged period of improvised forms of teaching and school absenteeism around the world [1]. Many potential consequences of the disruption of normal schooling have been discussed and investigated, including learning loss [2, 3], students’ feelings and mental health [4, 5], and students’ experiences and attitudes toward online learning [6, 7]. Little is known about the effects of the pandemic on students’ intelligence. It has been hypothesized that general increases in stress and anxiety during the pandemic limit cognitive functioning [8]. Moreover, academic achievement and intelligence have previously been shown to be highly interdependent [9], with strong positive effects of schooling on intelligence test performance [10, 11], This suggests that a prolonged disruption of regular schooling may also cause deficits in intellectual performance. In the present study, we therefore investigated the impact of the pandemic on intelligence test performance in a sample of German secondary school students. The results may provide some practical guidance on whether educational compensatory measures are needed and whether the consequences of the pandemic need to be considered in post-pandemic intelligence assessments.

### Schooling and intelligence

Schooling is a central predictor of many important outcomes, such as health [12], income [13], and intelligence [11]. Intelligence can be modeled as a hierarchy of multiple cognitive abilities of different generality [14]. Key aspects of intelligence are the capacities for information processing, problem solving, and abstract reasoning [15]. According to Linda Gottfredson [16 p13], “[Intelligence]… involves the ability to reason, plan, solve problems, think abstractly, comprehend complex ideas, learn quickly and learn from experience. It is not merely book learning, a narrow academic skill, or test-taking smarts. Rather, it reflects a broader and deeper capability for comprehending our surroundings—“catching on,” “making sense” of things, or “figuring out” what to do.” Crucially, intelligence, albeit being mostly discussed as a predictor of school achievement [17], has also been shown to be significantly impacted by schooling. For example, an increase in compulsory schooling in Norway from 7 to 9 years significantly increased the average intelligence quotient (IQ) [18]. The effect of one additional year of schooling was quantified by different studies between 1 and 10.8 IQ points [10, 11]. In a review, Rindermann [19] found an average positive effect of 5.6 IQ points per year of schooling in Germany. In a meta-analysis of 42 studies, Ritchie and Tucker-Drob [11] reported a benefit of 1 to 5 IQ points per year of schooling.

Based on these and other findings, Peng and Kievit [9] proposed a bidirectional perspective on the development of academic achievement and intelligence. They argued that in line with mutualism theory and the transactional model [20, 21], intelligence and academic achievement show positive reciprocal relations throughout development, leading to an increasingly strong association. Thus, any interventions targeting schooling should also affect intelligence [9]. A recent meta-analysis supported the suggested bidirectional longitudinal relations [21]. Schooling is hypothesized to be an important mechanism behind this bidirectionality [9]. During schooling, students invest their cognitive abilities to acquire academic skills and to perform academic tasks, which in turn involves the use of cognitive abilities; thus, schooling offers a long-term training for cognitive abilities. Sustained and high-quality schooling therefore should have direct positive effects on student’s academic and cognitive development as well as indirect effects by triggering cognitive-academic bidirectionality [9]. Further, students’ family socio economic status (SES) influences relations between students’ intelligence and academic achievement due to better early learning opportunities for children with a high family SES [21].

In light of the pandemic, the findings on the importance of schooling for intelligence development are troubling with regard to the global generation of students affected by prolonged school closures and irregular school attendance. Many researchers fear an increased number of school dropouts and reduced graduation rates in secondary education [22]. The expected effect of the pandemic on intelligence levels is difficult to quantify, as remote schooling does not equate to complete school absence. However, a lower quality of schooling by remote schooling for which teachers were not prepared in addition with a reduced time investment in education [2] over many months may still be very noticeable in intelligence test results.

### Schooling during the pandemic in Germany

**Fig 1:**
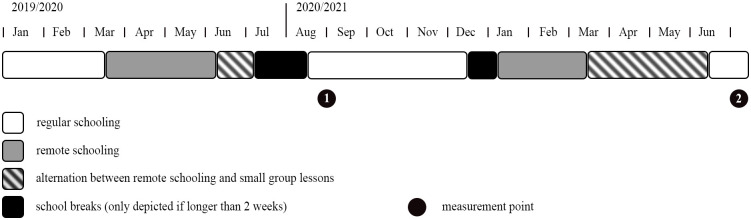
Timeline of schooling for students in Grades 7–10 in Rhineland-Palatine, Germany.

The present investigation was conducted with secondary school students from the German federal state of Rhineland-Palatinate. We first describe the school-related measures for the first six months of the pandemic (March-August 2020), leading up to the first measurement point. Second, we describe the measures for the next ten months, constituting the full 2020/2021 school year and leading up to the second measurement point. The full timeline for secondary school students is illustrated in Fig 1.

#### The 2019/2020 school year (second half)

On March 13, 2020, all schools closed by order of the state government [23]. On May 25, Grades 3 to 6 returned to school; on June 8, the rest of the students returned. During this time, students mostly alternated between remote schooling and small group lessons, as the number of students allowed in a classroom was limited to half class size [24]. The summer break was from July 6 to August 14, after which all students returned to school in mostly regular operation. Taken together, the majority of students did not attend school for three months while receiving assignments to be completed at home, returned to school under strict regulations or only in small groups for four weeks before going on a summer break for six weeks. During the three months of remote schooling, many students greatly reduced their time investment in education. On average, students spent only half of their usual time on daily educational activities, with 38% of secondary school students reporting less than 2 hours of schoolwork per day [25].

#### The 2020/2021 school year

The 2020/2021 school year started with regular schooling until December 16, when the Christmas break started early due to the pandemic situation [26]. After the break ended on January 4, remote schooling was conducted until February 14 for Grades 5 and 6 and until March 15 for Grades 7 to 13 [27, 28]. Students then alternated between remote schooling and small group lessons until June 14, when regular schooling returned [29]. Taken together, the students attended school regularly for four months, went on Christmas break for three weeks, did not attend school for three months while receiving assignments to be completed at home for ten weeks, returned to school under strict regulations or only in small groups for nine weeks, before returning to regular schooling.

#### The effects of the pandemic on students

The disruption of schooling and many other aspects of everyday life as well as the uncertainty and threatening nature of the pandemic situation have affected students psychologically in many ways. Camacho-Zuñiga et al. [4] investigated the emotional state of over 4,000 Mexican school and university students, finding low energy levels and negative feelings such as anxiety, stress, and tiredness to be prevalent during periods of lockdown. Similar results were found in other countries [30, 31]. Students’ feelings toward remote teaching were examined by Niemi and Kousa [7] in Finland, finding that online teaching was generally implemented successfully but also led to fatigue and loss in motivation in a portion of students. In addition to these emotional and motivational costs of the pandemic, many psychologists and educators also predicted a severe learning loss at the start of the pandemic [25, 32]. Students reported spending only approximately half the usual daily time on educational activities [25, 33]. This effect was especially strong in low-achieving students [33] and students from low-income families [34]. In line with earlier predictions, Engzell et al. [2] reported a learning loss of *d* = .08 after only eight weeks of lockdown in the Netherlands. In the US, gains in reading and math were 3 to 12 percentile points lower in the 2020–21 school year than in previous years [35]. Hammerstein et al. [3] reviewed the available literature on learning loss during the pandemic. They found that a majority of studies reported evidence for learning loss, with median effects of *d* = -.10 for math and *d* = -.09 for reading.

Early in the pandemic, Boals and Banks [8] warned that losses in cognitive performance were also to be expected in both children and adults. They argued that increases in stress and anxiety would cause mind wandering and worrying because of the constant stream of news on the issue and worry about oneself and others [36]. Mind wandering can be defined as thoughts about concerns that are unrelated to the task at hand and it takes up limited resources of executive functioning, potentially impairing any cognitive performance [37]. The pandemic brought numerous stressors for many students, such as increased tension at home, loss of social contact with peers, or worries related to safety and health; [38] further, it led to a loss of resources like physical activities, which help to reduce negative stress effects [39]. However, the effects of stress on cognitive performance are not well understood. There is some evidence that increased stress leads to lower cognitive functioning, but effects differed between different cognitive abilities and the research is largely based on acute stress instead of long-term elevated stress [40–43]. Thus, it is difficult to make precise predictions about the effects of pandemic-induced stress on cognitive performance on the basis of these findings.

Some studies have examined the effect of the pandemic on cognitive performance. Podlesek et al. [36] surveyed 830 Slovenian adults during the first wave and lockdown regarding their emotions (e.g., stress, anxiety, fatigue) and perceived changes in cognition. Participants reported mildly impaired cognitive functioning. The level of perceived impairment was significantly predicted by stress and negative emotions. Castanheira et al. [44] tested 1,517 American adults with a battery of executive functioning tasks and asked about pandemic-related worry and stress. They compared the results to a sample that was tested before the pandemic, finding that executive functioning was generally lower in the pandemic sample than in the prepandemic sample and that worry negatively predicted executive functioning in the pandemic sample. However, this research has so far been limited to adults; comparable findings for children and adolescents are missing.

### The present study

The COVID-19 pandemic and the associated disruption of regular schooling have negatively affected students in many ways. However, the impact on intelligence test performance has not yet been investigated. Previous studies [36, 44] focused on adult samples, were limited to self-reports or measures of executive functioning and only drew on cross-sectional data. Therefore, the present study investigated the intelligence test performance of German secondary school students during the pandemic. Like most other research on the effects of the pandemic, we faced some challenges. The unpredictability of the pandemic precluded the anticipatory launch of a longitudinal study with measurement time points prior to the onset of the pandemic. In addition, the uniform impact of the pandemic on all students prevented the use of a control group design. We compensated for these issues in two ways. First, two prepandemic samples were available that had been tested with the same intelligence test. These samples were used to create highly comparable comparison groups using propensity score matching. Second, the ongoing effects of the pandemic were assessed by retesting the pandemic sample after one full school year.

The pandemic sample assessed in 2020 comprised students from regular classes and special classes for gifted students. We conducted two sets of analyses. First, we compared the intelligence test results of the pandemic sample tested in August or September 2020 to the results of two comparable samples tested in 2002 and 2012. In Analysis 1a, we compared two propensity score matched subsamples from the pandemic 2020 sample and the prepandemic 2002 sample, comprising students from both regular classes and special classes for the gifted. The 2002 data stem from the norming sample of the intelligence test used. In Analysis 1b, we compared three propensity score matched subsamples from the pandemic 2020 sample and the prepandemic 2012 and 2002 samples. Samples in Analysis 1b only comprised students from special classes for the gifted because the 2012 sample did not include students from regular classes. The 2020–2002 comparison (Analysis 1a) is therefore more representative for the entire student body. By comparing the 2020, 2012, and 2002 samples (Analysis 1b), we investigated if any observed differences in Analysis 1a are better interpreted as part of a more continuous development of test scores from 2002 to 2012 to 2020. We expected to find significantly lower intelligence scores for the 2020 sample in both analyses and no decreasing trend between 2002 and 2012, as no decreasing intelligence levels have generally been observed during this time in Germany [45].

In Analysis 2, we investigated the mean level change in intelligence after one school year in the pandemic 2020 sample (retest in 2021). Perceived stress during this school year was also assessed. We expected a decline in intelligence test scores when taking typical retest effects into account. That is, we expected a decline in scores or at least smaller increases than the positive retest effects expected based on meta-analytic evidence on retest effects in intelligence testing [46]. Furthermore, based on the predictions made by Boals and Banks [8], we investigated whether the level of perceived stress could explain changes between the two measurements. That is, we investigated if stress was a significant negative predictor of latent change scores of cognitive abilities.

## Methods and materials

### Participants

#### 2020 sample (pandemic)

A total of 424 students from Grade 7 (34.67%), 8 (33.25%), and 9 (32.08%) were tested in late August or early September 2020 with the Berlin Structure-of-Intelligence Test (BIS-HB) [47]. The students attended either regular classes or special classes for the gifted (45.75%) (schools offered both class types) in four German grammar schools in Rhineland-Palatine. The mean age was 13.34 years (*SD* = .99), and 41.98% of the sample identified as female. Of the sample, 98 students were too young or too old to receive a norm-referenced IQ score from the intelligence test (norms for ages 12.5–16.5 years) and were excluded from the analyses. Note that for additional 24 participants, some of the intelligence scales were not available because they did not complete some of the corresponding tasks according to the instructions or were absent for a short period of time during the testing. These 24 students with missing values did not significantly differ from the 302 students without missing values regarding age (mean age = 13.66 vs. 13.80 years; *T* = .69, *p* = .499), gender (missing percentage: 8.00% of males vs. 6.60% of females; *Chi*^*2*^ = .23, *p* = .631), and grade level (missing percentage: 7.80% of Grade 7 vs. 6.80% of Grade 8 vs. 7.00% of Grade 9; *Chi*^*2*^ = .29, *p* = .962). However, missing values were related to class type (*Chi*^*2*^ = 5.58, *p* = .018): Students from regular classes were more likely to have missing scores than students form special classes for the gifted (missing percentage: 10.40% vs. 3.50%). We only excluded students with missings on all variables from the analyses. Sample sizes and demographic variables for the unmatched and matched samples are presented in Table 1.

**Table 1:** Demographic information for the unmatched and matched samples.

Sample	*N*	Age	% Female	% Gifted Class	% Grade 7	% Grade 8	% Grade 9
*unmatched*							
2020	424	13.34 (.99)	41.98	45.75	34.67	33.25	32.08
2012	197	13.87 (.58)	41.62	100.00	0.00	100.00	0.00
2002	1506	14.54 (1.35)	44.08	29.88	28.29^a^	24.50^a^	22.31^b^
*Analysis 1a matched samples*							
2020	104	13.64 (.84)	45.19	26.92	34.62	31.73	33.65
2002	104	13.65 (.84)	45.19	26.92	34.62	31.73	33.65
*Analysis 1b matched samples*							
2020	110	13.74 (.60)	35.25	100.00	10.00	41.80	48.20
2012	110	13.76 (.59)	33.06	100.00	0	100.00	0
2002	110	13.76 (.59)	33.06	100.00	50.00	42.70	7.30

*Note*.

^a^ = students attended a special class for the gifted (selection of students for these classes is based on intelligence tests, school achievements, and teacher observations in trial lessons).

^b^ = 24.9% of the sample attended Grades 5, 6, or 10.

Of the 326 students with an IQ score at the first measurement point, 257 (78.83%) were retested in July 2021. The 69 students missing at the second time of measurement did not significantly differ from the 257 students that were retested with regard to age (mean age = 13.67 vs. 13.61 years; *T* = .55, *p* = .581), gender (missing percentage: 21.50% males vs. 21.20% females; Chi^2^ = 1.90, *p* = .387), class type (missing percentage: 22.50% regular classes vs. 19.40% gifted classes; Chi^2^ = 4.58, *p* = .499), and grade level (missing percentage: 23.40% Grade 7 vs. 19.70% Grade 8 vs. 21.50% Grade 9; Chi^2^ = .38, *p* = .827).

Note that for additional 17 participants, some of the intelligence scales were not available at T2 because they did not complete the corresponding tasks correctly or were absent for a short period of time during the testing. These 17 students did not significantly differ from students without any missing values at T2 regarding age (mean age = 13.61 vs. 13.88 years; *T* = 1.35, *p* = .179), gender (missing percentage: 6.90% males vs. 3.70% females; Chi^2^ = 2.05, *p* = .359), class type (missing percentage: 6.90% regular classes vs. 3.50% gifted classes; Chi^2^ = .19, *p* = .186), and grade level (missing percentage: 4.30% Grade 7 vs. 5.60% Grade 8 vs. 5.80% Grade 9; Chi^2^ = .22, *p* = .896). We only excluded students with missings on all variables.

All parents of the participants gave written informed consent in accordance with the Declaration of Helsinki. The protocol was approved by the principals of the participating schools. The data collections were approved by the Supervision and Services Directorate of Rhineland-Palatinate on the basis of ethical and data protection requirements (Aufsichts- und Dienstleistungsdirektion; protocol numbers 153–20 and 226–21).

#### 2012 sample (prepandemic)

A total of 197 Grade 8 students who attended the same four schools as the 2020 sample were tested between 2011 and 2013 (“2012 sample” for short) with the BIS-HB. All students attended special classes for the gifted. The mean age was 13.87 years (*SD* = .48), and 41.42% of the sample identified as female. Sample sizes and demographic variables for the full sample and matched samples are presented in Table 1. There were no missing data. All parents of the participants gave written informed consent in accordance with the Declaration of Helsinki. The protocol was approved by the principals of the participating schools. The data collection was approved by the Supervision and Services Directorate of Rhineland-Palatinate on the basis of ethical and data protection requirements (Aufsichts- und Dienstleistungsdirektion; protocol number 32–03 405/29/05).

#### 2002 sample (prepandemic)

1506 Grade 5 to 10 students attending schools in five German federal states were tested in 2002 in the context of the BIS-HB standardization [47]. These students were distributed across all German school tracks, with a subset of 571 students attending regular classes in grammar schools and 450 students attending special classes for the gifted in grammar schools. The mean age was 14.54 years (*SD* = 1.35), and 44.62% of the sample identified as female. Sample sizes and demographic variables for the full sample and matched samples are presented in Table 1. There were no missing data. All parents of the participants gave written informed consent in accordance with the Declaration of Helsinki. The protocol was approved by the principals of the participating schools. The data for this study was collected by order of and in accordance with the recommendations of the German Federal Ministry of Education and Research. The present study represents a secondary analysis of this dataset.

### Measures

#### Intelligence

**Fig 2:**
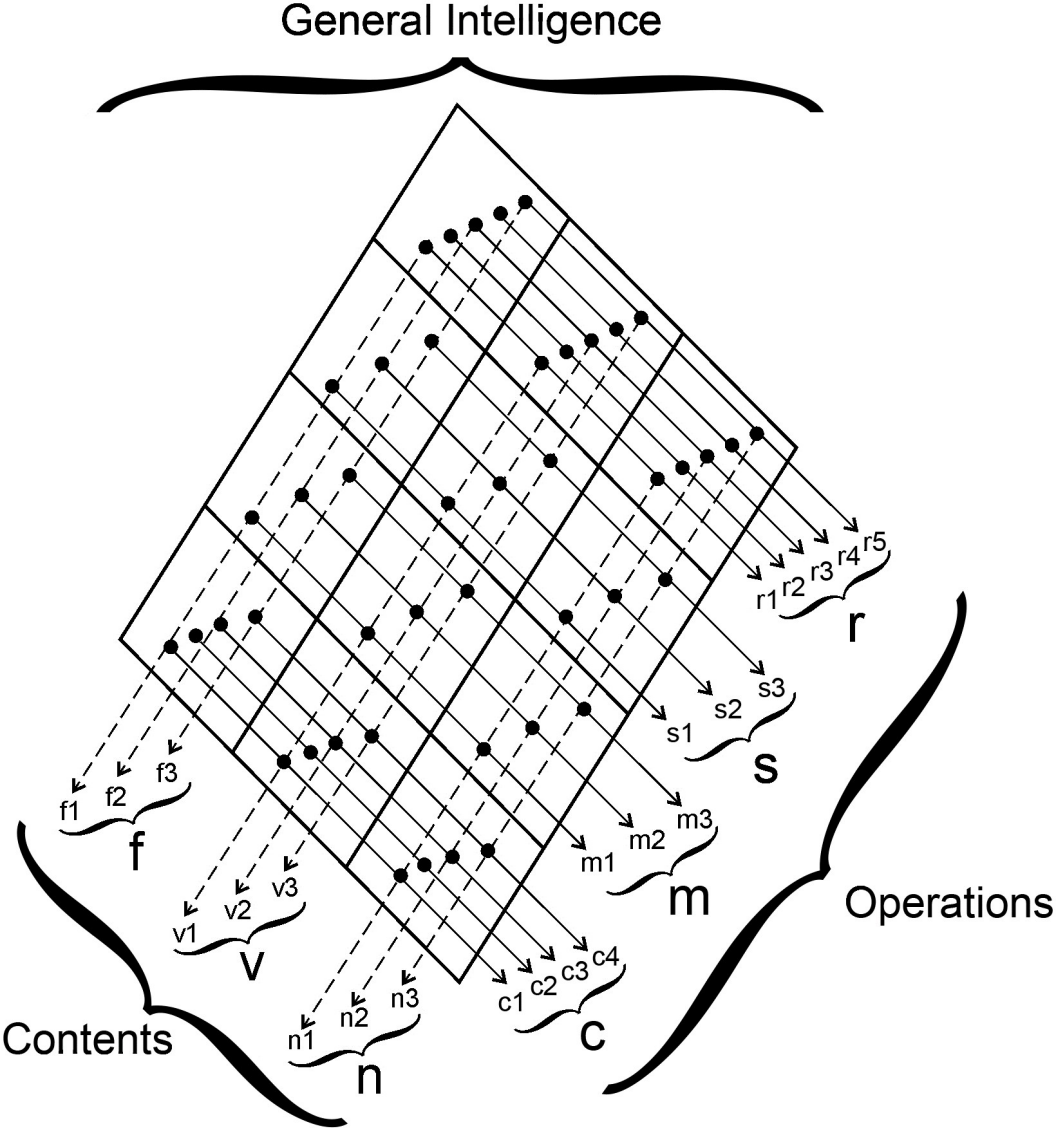
Structure of the Berlin model of Intelligence Structure (BIS). f = Figural Ability, v = Verbal Ability, n = Numerical Ability, r = Reasoning, s = Processing Speed, m = Memory, c = Creativity.

The BIS-HB [35] is a paper-and-pencil intelligence test designed to capture the intelligence structure of above-average and high-ability adolescents. It can also be applied for testing average and below-average ability individuals. The test is based on the Berlin Model of Intelligence Structure (BIS) by Jäger [48]. The BIS is a faceted model of intelligence (Fig 2). The operation facet includes processing speed [S], memory [M], creativity [C], and reasoning [R]. The content facet includes verbal [V], numerical [N], and figural [F] ability. Each individual test item is assigned to a combination of one operation and one content (e.g., a verbal processing speed task). Thus, each operation score is a combination of verbal, numerical, and figural tasks of the respective operation, and each content score is a combination of speed, memory, creativity, and reasoning tasks of the respective content. On a higher hierarchical level, the abilities from the operation facet and from the content facet are integrated into general intelligence. The BIS-HB comprises 45 tasks assessing the four operations and three domains, providing eight test scores (S, M, C, R, V, N, F, and *g*). The test was used in all samples and at both measurement points of the pandemic sample.

The construct validity of the BIS-HB has been documented by confirmatory factor analyses (multiple group comparisons for the different age and ability groups; range of CFIs = .97-.99); criterion validity has been documented by correlations with other intelligence tests (e.g., BIS-HB reasoning with the German version of the culture fair test [49]: *r* = .74, *N* = 1080; BIS-HB creativity with a verbal creativity test [VKT; 50]: *r* = .52, *N* = 146) and school grades (BIS-HB IQ with grade point average: *r* = .50, *N* = 1320; BIS-HB reasoning with grade point average in Math and sciences: *r* = .47, *N* = 1313) [47]. The operations of the BIS are conceptually close to corresponding abilities in the Cattell-Horn-Carroll model (CHC-model) [14]. Processing speed is included in both models, memory is similar to learning efficiency in the CHC-model, creativity to retrieval fluency, and reasoning to fluid reasoning. This proximity to another established structural model of intelligence makes it unlikely that the results of the current study are limited to the scales of the BIS.

#### Perceived stress

At the second measurement point of the pandemic sample, we included a three-item scale assessing the perceived stress and changes in well-being caused by the disruption of regular schooling and the pandemic. The scale was adapted from the School Barometer in Germany, Austria and Switzerland, [51] and the answer format was a 5-point Likert scale. The three questions were as follows (translation by authors):

1. I was doing well with the school closures.
2. I was doing well with the alternating lessons.
3. I find the "coronavirus situation" stressful.

### Analyses

#### Analysis 1

We conducted two separate analyses, one comparing the 2020 sample to the 2002 sample (Analysis 1a) and one comparing the 2020 sample to both the 2012 and 2002 samples (Analysis 1b). Before each analysis, we conducted propensity score (PS) matching to create comparable subsamples. Then, we tested the differences in intelligence between the resulting subgroups using MANOVA, ANOVA, and Discriminant Function Analyses.

*Propensity score matching*. We used PS matching with the matchIt package in R to control for demographic differences between the 2020 sample and the 2002 sample (Analysis 1a) and between students from special classes for the gifted from the 2020 sample, the 2012 sample, and the 2002 sample (Analysis 1b). Propensity score matching is a method in which individuals from one group are matched to individuals from a second group based on the calculated PS for each person [52]. The PS represents the probability of assignment to a particular group based on a vector of observed covariates [53]. Thus, by ensuring that two groups do not differ in their PS, one controls for potential a priori differences between the groups on the observed covariates.

In Analysis 1a, students from the 2002 sample were matched to students from the 2020 sample using the nearest neighbor algorithm [54], based on age as a continuous covariate as well as sex, grade level, and class type that had to match exactly. In Analysis 1b, students from regular classes were excluded from the 2020 and 2002 samples because no such students were available in the 2012 sample. In two separate matching procedures, students from the 2020 sample and then students from the 2002 sample were matched to students from the 2012 sample using the nearest neighbor algorithm, based on age as a continuous covariate as well as sex that had to match exactly.

In all conducted PS matching procedures, we applied the recommended caliper of .20 [55] and allowed the algorithm to discard cases from both groups. As a criterion to evaluate the quality of the PS matchings we calculated Hedges’ *g* effect sizes for differences of the propensity score and age between the matched samples (Hedges’ $g=\frac{M1-M2}{SD\_pooled}$). A Hedges’ *g* smaller than .20 was interpreted as a negligible difference. In addition, we conducted the overall balance test [56] and L1 statistics [57] that both test whether the matched samples differ on all covariates combined. L1 values can range from 0 to 1 with 1 indicating a total imbalance between the samples and 0 indicating zero differences between the matched samples.

*ANOVA*, *MANOVA and discriminant function analysis*. In both Analyses 1a and 1b, we performed a multivariate analysis of variance (MANOVA) using the seven BIS-HB specific ability scores as dependent variables. In case that one MANOVA indicated a significant multivariate main effect, we conducted a discriminant function analysis as a post hoc test to find out which particular intelligence scales discriminate between the samples. Note that the BIS-HB *g*-factor was excluded from the MANOVA because it represents the sum of all specific ability scores. We conducted one additional ANOVA including the BIS-HB *g*-factor as the dependent variable. As a precondition for MANOVA we conducted several tests. First, for multivariate normality [58], we estimated Marida’s multivariate skewness and kurtosis [59] and Royston’s extension of the Shapiro–Wilk test [60]. Second, to identify multivariate outliers, we obtained Mahalanobis distances [61] and calculated the respective Chi-squared test for each participant. Third, the Levene test [62] for equality of variances and the Box-test [63] for equality of covariance matrices were conducted. In case of a significant violation of the preconditions of MANOVA, we conducted non-parametric robustness check analyses in the form of permutation-based multivariate analysis of variance (PERMANOVA) using the R package ‘vegan’ [64]. As preconditions for ANOVA, we tested the homogeneity, homoskedasticity, and the univariate normality of the *g*-factor by the Levene test, the Breusch Pagan test, and the Kolmogorov-Smirnov test, respectively. In case of a significant violation of any preconditions, we conducted non-parametric robustness check analyses in the form of the Kruskal-Wallis test in SPSS.

Analysis 2. *Mean-level change*. We investigated the effect size and statistical significance of mean-level change in all eight BIS-HB ability scores by computing Hedges’ g [65]. We compared the results to the average retest effects reported in a recent meta-analysis [46].

**Fig 3:**
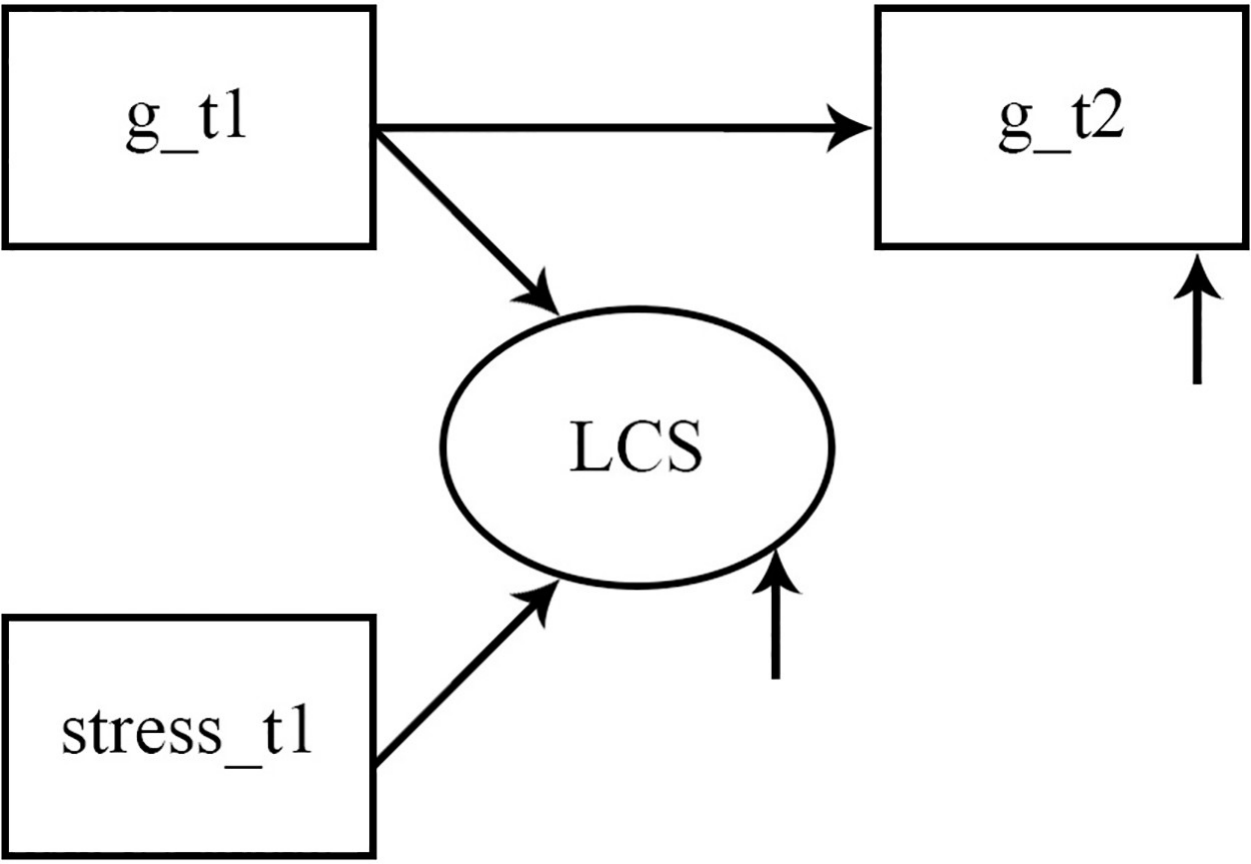
Latent change score structure equation models in Study 2. LCS = Latent change score. g_t1 = General Intelligence at the first measurement point. g_t2 = General Intelligence at the second measurement point.

*Latent change score analysis*. We calculated eight latent change score (LCS) structural equation models (SEM) to test whether perceived stress significantly predicted changes in the BIS-HB ability scores. All SEMs were calculated in Mplus version 8.4 [66] by using the maximum likelihood estimator with robust standard errors (MLR) [67]. We applied the “type is complex” option to account for the nested data structure (“students within classes”). Missing data was handled by using the full information maximum likelihood algorithm [66]. In each SEM, a latent change score of either the general intelligence or one specific ability score was estimated based on the T1 and T2 measurements of intelligence. As an example, we present the SEM based on general intelligence in Fig 3. The T1 intelligence measure as well as the LCS predict the T2 intelligence measure with a fixed value of 1. That is, the T2 measure is completely determined by the first measure and the change value. The LCS is a latent variable that depicts interindividual differences in the change in students’ intelligence between T1 and T2. In each SEM, perceived stress was included as a predictor of the LCS. That is, interindividual differences in the intraindividual intelligence change over time were predicted by students’ perceived stress.

## Results

### Analysis 1a: 2002 vs. 2020

**Fig 4:**
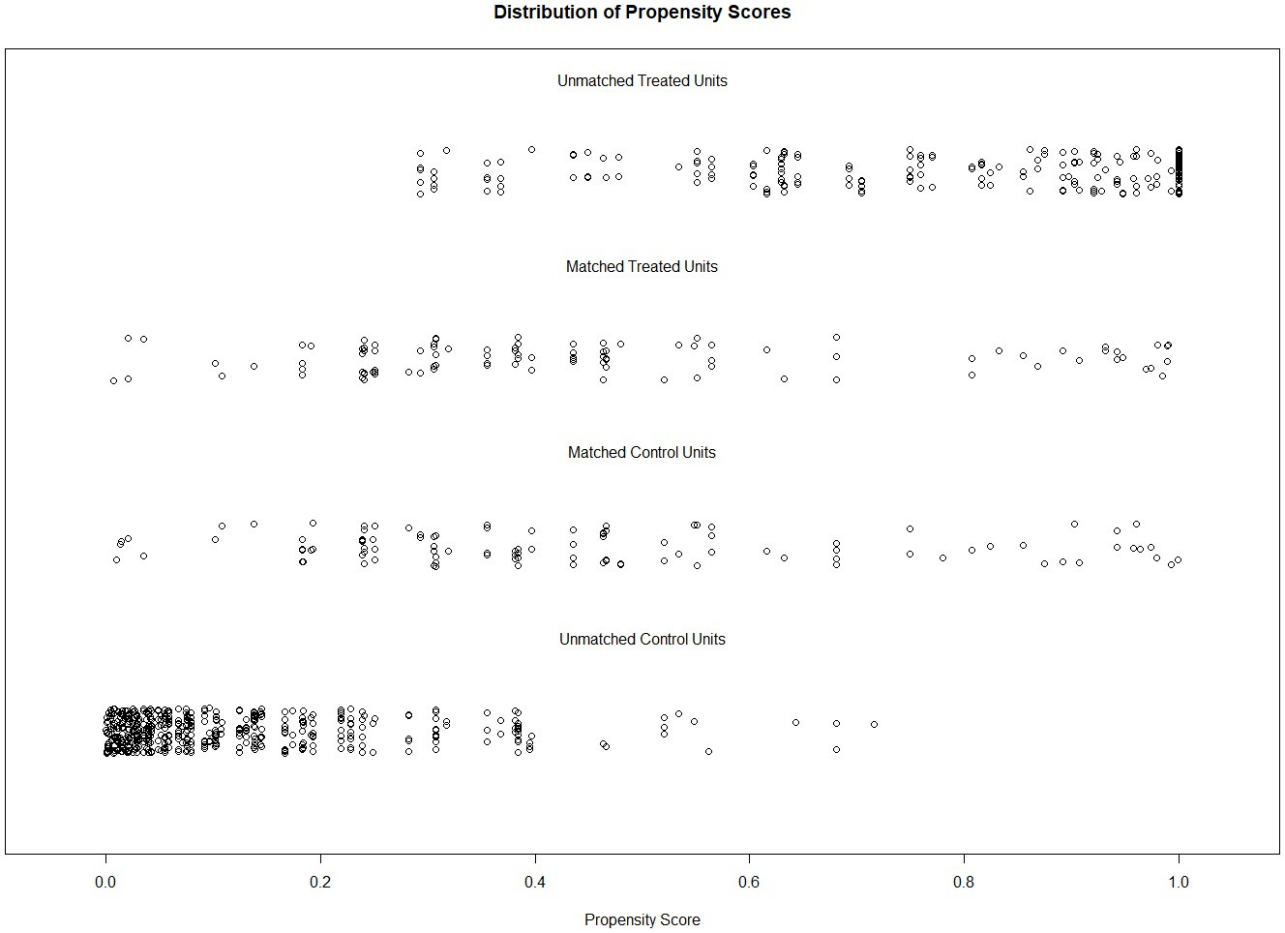
Propensity score distribution in Analysis 1a for the overlap between the 2002 sample and the 2020 sample. Unmatched units were discarded after the matching procedure to ensure an optimal overlap in propensity scores of the two groups.

The PS matching algorithm in Analysis 1a matched 104 students from the 2002 sample to 104 students from the 2020 sample. Descriptive differences on all covariates before and after the PS matching are presented in Table 1. The matched samples showed exact same proportions of gender, class type, and grade level, as well as negligible differences in age (mean age = 13.64 vs. 13.65 years; Hedges’ *g* = .01) and PS (mean PS = .46 vs. .45; Hedges’ *g* = .04). Hedges’ *g*, percentage of the propensity score overlap, the overall balance test, and L1 statistics before and after the matching procedure are reported in Table 2. The PS distribution of the two groups before and after matching is depicted in Fig 4.

**Table 2:** Balance tests of the matched samples.

Sample	*Percentage of matched units in samples*			Hedges’ *g*		*Overall balance test*			*Multivariate balance measure L1*	
				PS	Age	χ^2^	*df*	*p*		
*Analysis 1a*										
2020 vs. 2002	24.53% (2020)	6.91% (2002)	10.78% (overall)	.04	.01	.02	5	1	.67 (unmatched)	.10 (matched)
*Analysis1 b*										
2020 vs. 2012	25.94% (2020)	55.84% (2012)	15.51% (overall)	0	.03	.05	2	.977	.58 (unmatched)	.04 (matched)
2020 vs. 2002	25.94% (2020)	7.30% (2012)		0	.04	.07	2	.963	.79 (unmatched)	.04 (matched)
2012 vs. 2002	55.84% (2012)	7.30% (2012)		0	.02	0	2	.988	.57 (unmatched)	.05 (matched)

*Note*. PS = Propensity Score.

**Fig 5:**
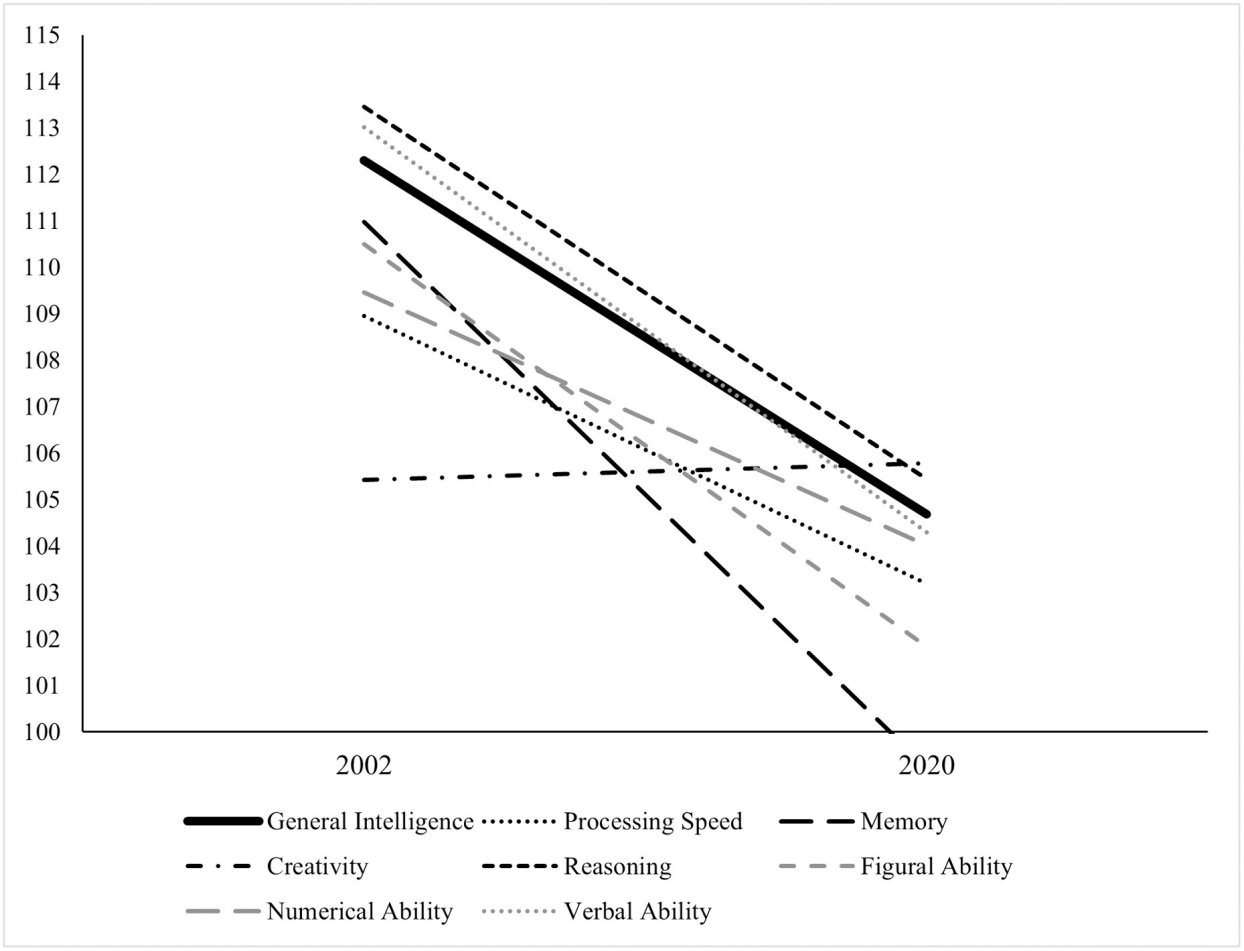
Mean IQs for the different ability scores in Analysis 1a.

The BIS-HB ability score means for the matched 2002 and 2020 samples are presented in Table 3 and Fig 5. The MANOVA revealed a large, statistically significant difference between the samples in their BIS-HB results in favor of the matched 2002 sample (*F*[7, 200] = 6.881, *p* < .001, partial η^2^ = .194). The discriminant function analysis indicated a significant function (i.e., Function 1) that differentiated between the 2002 sample and the 2020 sample (Eigenwert = .26, Wilks-Lambda = .80, Chi^2^ = 46.016, *df* = 7, *p* < .001). The structural coefficients of this function are presented in Table 4. All intelligence scales except for creativity showed substantial structural coefficients (*r* ranged from .38 in N to .79 in M) indicating meaningful differences on these scales between the 2002 sample and the 2020 sample in the favor of the 2002 sample. Creativity indicated virtually no relation (*r* = -.02) with the calculated function and thus did not discriminate between the samples. Finally, the ANOVA revealed a medium difference between the samples in the *g*-factor in favor of the matched 2002 sample (*F*[1, 206] = 15.677, *p* < .001, partial η^2^ = .071).

**Table 3:** Means (and *SD*) of the different ability scores in Analysis 1a.

Sample	*g*	S	M	C	R	F	N	V
2002	112.30 (14.55)	108.95 (13.20)	110.98 (15.26)	105.43 (14.48)	113.45 (15.03)	110.5 (15.91)	109.46 (14.79)	113.01 (13.10)
2020	104.68 (13.15)	103.19 (13.21)	99.24 (14.30)	105.78 (13.71)	105.44 (13.59)	103.95 (13.57)	104.01 (13.53)	104.29 (14.14)

*Note*. *g* = General Intelligence, S = Processing Speed, M = Memory, C = Creativity, R = Reasoning, F = Figural Ability, N = Numerical Ability, V = Verbal Ability

**Table 4:** Structure coefficients of the discriminant function 1 in Analysis 1a.

Variable	*r*
Processing Speed S	.43
Memory M	.79
Creativity C	-.02
Reasoning R	.56
Figural Ability F	.44
Numerical Ability N	.38
Verbal Ability V	.64

Marida’s multivariate skewness and kurtosis tests indicated a significant violation of the multivariate normality in the MANOVA (Marida’s skewness = 633.70, *p* < .001; Marida`s kurtosis = 18.41, *p* < .001). Royston’s test indicated no significant violation of the multivariate normality (H = 9.12, *p* = .167). Chi-squared tests based on Mahalanobis distances indicated eleven significant outliers (*p* > .05) that were checked and not attributed to coding errors. The robustness check in the form of PERMANOVA supported the MANOVA results, indicating a significant difference between the groups (R^2^ = .06, *p* = .002).

The Levene test (*F* based on means = .74, df = 206, *p* = .392), the Breusch Pagan test (Chi-square = .95, df = 1, *p* = .330), and the Kolmogorov-Smirnov test (K = .06, df = 208, *p* = .200) indicated no violation of the homogeneity, homoskedasticity, and normality of the *g*-factor, respectively. Therefore, no non-parametric robustness check analyses were conducted in addition to the ANOVA.

### Analysis 1b: 2002 vs. 2012 vs. 2020

**Fig 6:**
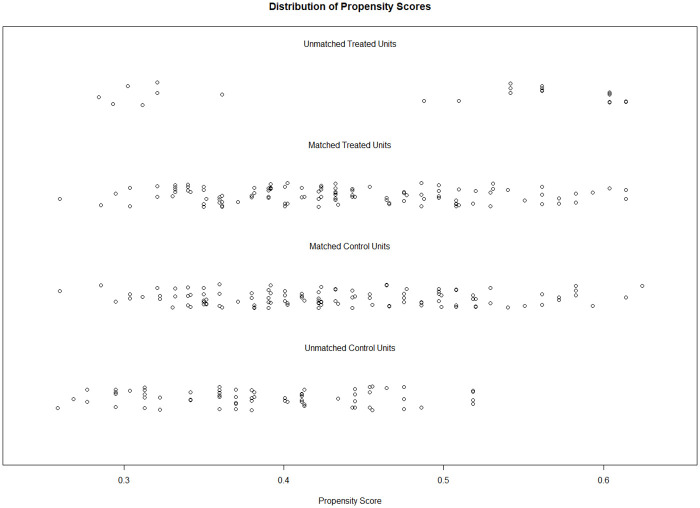
Propensity score distribution in Analysis 1b for the overlap between the 2012 sample and the 2020 sample. Unmatched units were discarded after the matching procedure to ensure an optimal overlap in propensity scores of the two groups.

**Fig 7:**
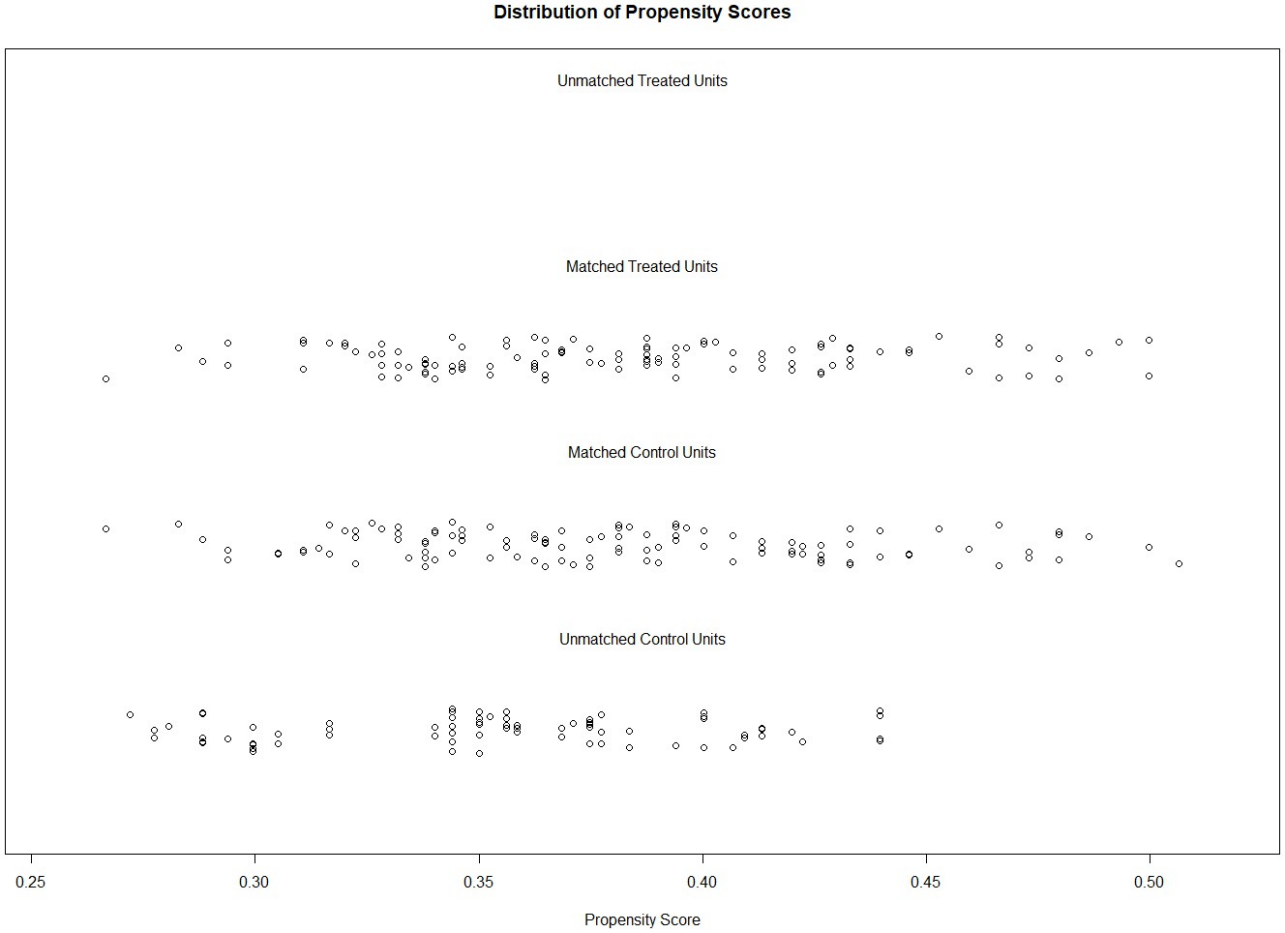
Propensity score distribution in Analysis 1b for the overlap between the 2002 sample and the 2020 sample. Unmatched units were discarded after the matching procedure to ensure an optimal overlap in propensity scores of the two groups.

The PS matching algorithm matched 113 students from the 2012 sample to 113 students from the 2020 sample. A second PS matching algorithm matched 110 students from the sample 2002 to the 110 students from the 2012 sample. Finally, a third PS matching procedure matched 110 students from the 2012 sample that were chosen by the second PS matching to 110 students from 2020 sample (i.e., three students from the first PS matching were discarded because they did not have a match in the second PS matching). Descriptive differences on all covariates before and after the PS matching are presented in Table 1. The matched samples showed the same proportions in gender and class type, as well as negligible differences in age (mean age = 13.74 vs. 13.76 vs. 13.76 years; average Hedges’ *g* = .03) and PS (mean PS = .43 vs. .43 vs. 43; average Hedges’ *g* = 0). They differed in grade level as only Grade 8 students were available in the 2012 sample and the covariate could therefore not be taken into account in the matching procedure. The PS distribution of the first and the second PS matchings in Analyses 1b are presented in Figs 6 and 7, respectively.

**Fig 8:**
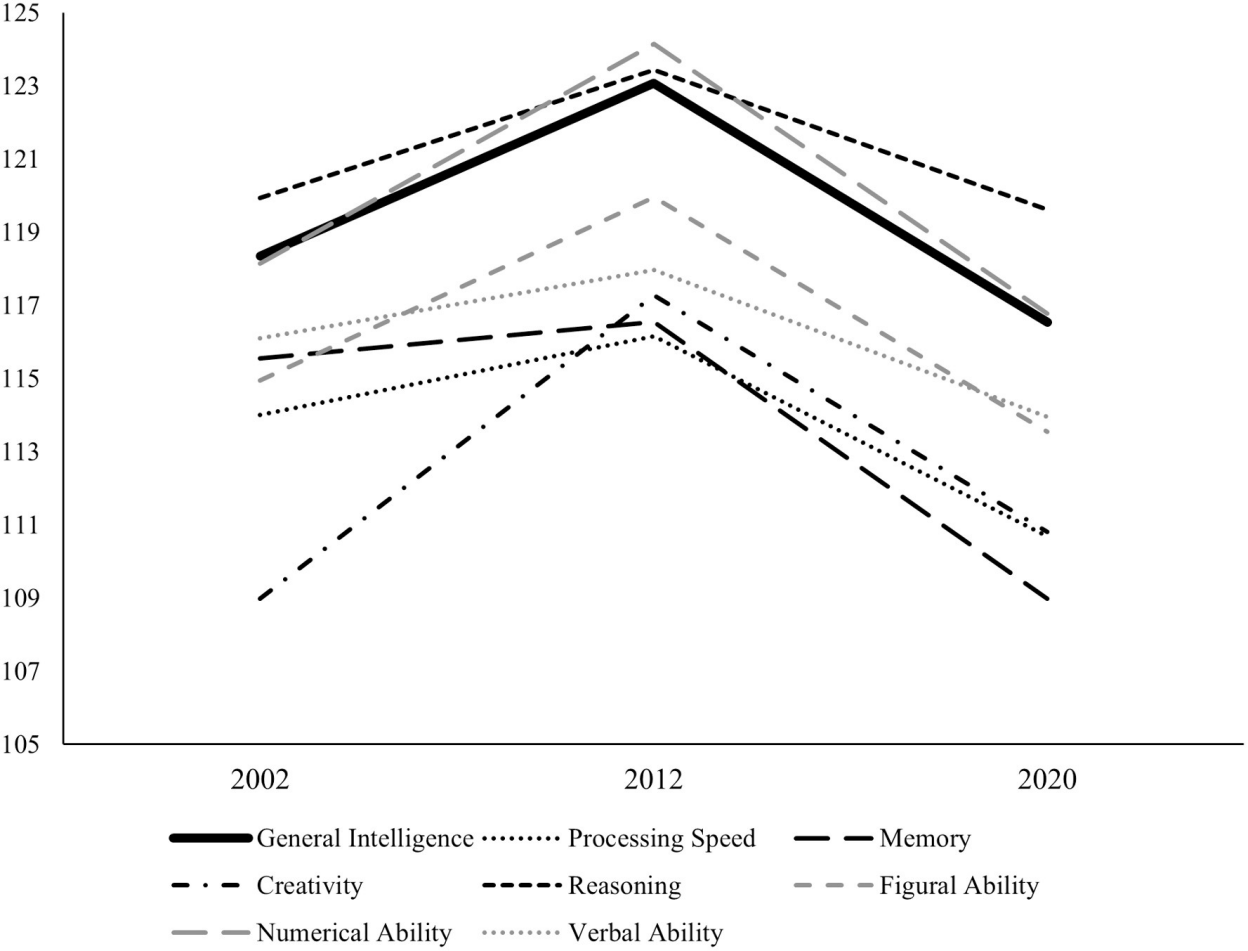
Mean IQs for the different ability scores in Analysis 1b.

The BIS-HB ability score means for the matched 2002, 2012, and 2020 samples are presented in Table 5 and Fig 8. The MANOVA revealed a medium-sized, statistically significant difference between the samples in their BIS-HB results (*F*[14, 644] = 4.409, *p* < .001, partial η^2^ = .087). The discriminant function analysis indicated two significant functions that differentiated between the 2002 sample, the 2012 sample, and the 2020 sample (Function 1: Eigenwert = .12, Wilks-Lambda = .83, Chi^2^ = 59.41, *df* = 14, *p* < .001; Function 2: Eigenwert = .08, Wilks-Lambda = .93, Chi^2^ = 24.03, *df* = 6, *p* < .001). Function 1 and Function 2 explained 60% and 40% of the variance, respectively. The structural coefficients of both functions are presented in Table 6. In Function 1, all intelligence scales showed substantial structural coefficients (*r* ranged from .31 in N to .87 in V). This function discriminated between the 2012 sample on one hand and between the 2002 sample and 2020 sample on the other hand indicating higher intelligence scores in the 2012 sample than in the other two samples. In Function 2, all intelligence scales showed substantial structural coefficients (*r* ranged from .30 in F to .82 in M) except for C (*r* = -.04) and R (*r* = .17). Function 2 mainly differentiated between the 2002 sample and the 2020 sample indicating higher intelligence scores in the 2002 sample. Finally, the ANOVA revealed a medium difference between the samples in the *g*-factor score (*F*[2, 327] = 12.359, *p* < .001, partial η^2^ = .070). Tukey post-hoc-tests indicated higher *g*-factor scores in the 2012 sample than in the 2002 sample (*p* = .002) and the 2020 sample (*p* < .001). The 2002 sample and the 2020 sample did not differ significantly in the *g*-factor score (*p* = .375).

**Table 5:** Means (and *SD*) of the different ability scores in Analysis 1b.

Sample	*g*	S	M	C	R	F	N	V
2002	118.35 (11.18)	114.00 (11.20)	115.55 (13.24)	108.98 (13.17)	119.93 (10.89)	114.95 (13.06)	116.11 (11.60)	118.15 (10.60)
2012	123.08 (10.21)	116.16 (12.97)	116.55 (13.81)	117.27 (13.18)	123.43 (8.24)	119.68 (11.69)	117.93 (11.77))	124.14 (10.58)
2020	116.54 (8.70)	110.69 (11.69)	108.98 (12.53)	110.81 (13.80)	119.62 (8.12)	113.54 (11.39)	113.95 (10.43)	116.78 (9.39)

*Note*. *g* = General Intelligence, S = Processing Speed, M = Memory, C = Creativity, R = Reasoning, F = Figural Ability, N = Numerical Ability, V = Verbal Ability

**Table 6:** Structure coefficients of the discriminant Function 1 and Function 2 in Analysis 1b.

	*r*	
Variable	Function 1	Function 2
Processing Speed S	.38	.50
Memory M	.34	.82
Creativity C	.79	-.04
Reasoning R	.54	.17
Figural Ability F	.59	.30
Numerical Ability N	.31	.36
Verbal Ability V	.87	.39

Marida’s multivariate skewness and kurtosis tests indicated a significant violation of the multivariate normality (Marida’s skewness = 248.64, *p* < .001; Marida`s kurtosis = 5.87, *p* < .001). Similarly, Royston’s test indicated a significant violation of the multivariate normality (H = 26.68, *p* < .001). Chi-squared tests based on Mahalanobis distances indicated 12 significant outliers (*p* > .05) that were checked and not attributed to coding errors. In addition, the Levene test for equality of variances and the Box-test for equality of covariance matrices were significant (*p* < .05). We therefore again conducted a PERMANOVA as a robustness check. The results were similar to the MANOVA, also indicating a significant difference between the groups (R^2^ = .02, *p* = .001). Post hoc tests based on the custom R script ‘pairwiseAdonis’ (https://github.com/pmartinezarbizu/pairwiseAdonis) indicated that all three groups differed significantly from one another (2002 vs. 2012: R^2^ = .04, *p* = .003; 2002 vs. 2020: R^2^ = .02, *p* = .048; 2012 vs. 2020, R^2^ = .06, *p* = .003; all *p*-values Bonferroni-corrected).

The Levene test indicated a violation of the homogeneity in the *g*-factor between the samples (*F* based on means = 5.12, df = 328, *p* = .024). The Breusch Pagan test indicated a violation of the homoskedasticity in the *g*-factor between the samples (Chi-square = 4.89, df = 1, *p* = .027). The Kolmogorov-Smirnov test indicated no violation of normality of the *g*-factor (K = .05, df = 330, *p* = .200). We therefore conducted a the Kruskal-Wallis test as a robustness check for the ANOVA. The results were similar to the ANOVA. We observed a significant main effect (*F* = 23.67, df = 2, p < .001) and the Bonferroni post-hoc-tests indicated higher *g*-factor scores in the 2012 sample than in the 2002 sample (*p* = .003) and the 2020 sample (*p* < .001). The 2002 sample and the 2020 sample did not differ significantly in the *g*-factor score (*p* = .461).

### Analysis 2: 2020/2021

#### Mean-level change

**Fig 9:**
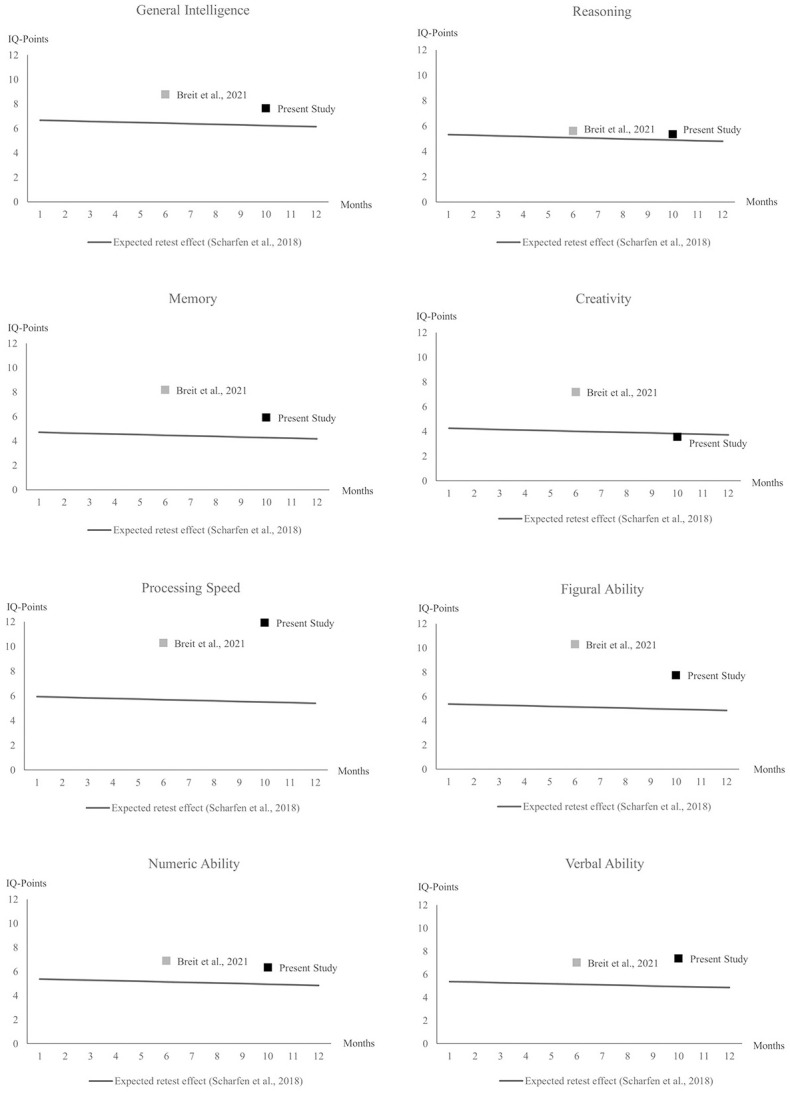
Observed mean-level change in the present study (2020 sample) and the BIS-HB standardization subsample reported in Breit et al., 2021 [75].

Table 7 shows the mean level change in all BIS-HB ability scores. All scores significantly increased from test to retest. Test-retest correlations ranged from *r* = .71 (Memory) to *r* = .87 (General Intelligence). The median increase was 6.86 IQ points (Hedges’ *g* = .53), ranging from 3.56 IQ points for Creativity (Hedges’ *g* = .22) to 11.93 IQ points for Processing Speed (Hedges’ *g* = .90). General Intelligence increased by 7.56 IQ points (Hedges’ *g* = .59). Fig 9 graphically compares the observed mean-level change to typical mean-level change observed meta-analytically and in a previous investigation using BIS-HB ability scores. There was neither a remarkable decrease in the intelligence test scores over the 2020–2021 school year nor a strong increase that may be interpreted as “catching up” to previous cohorts, as indicated by largely comparable retest effects to the previous investigation and the meta-analysis.

**Table 7:** Means and standard deviations of BIS-HB specific ability scores across a test–retest interval of ten months.

	Test		Retest			
Ability Score	*M*	*SD*	*M*	*SD*	Difference	Hedges’ *g*
General Intelligence	108.78	12.33	116.43	13.65	7.65	.589
Reasoning	109.72	12.93	115.08	13.89	5.36	.399
Memory	102.35	14.11	108.27	16.36	5.92	.387
Creativity	106.77	14.39	110.33	17.40	3.56	.223
Processing Speed	105.09	12.78	117.02	13.62	11.93	.903
Figural Ability	106.23	13.96	113.99	16.11	7.76	.515
Numeric Ability	106.42	12.96	112.77	13.84	6.35	.474
Verbal Ability	108.98	12.52	116.34	12.35	7.36	.592

*Note*. All differences were *p* < .001 after applying Bonferroni correction.

#### Latent change score analysis

LCS SEMs indicated that interindividual differences in the intraindividual change of BIS-HB ability scores were not predicted by perceived stress. The standardized parameters for each ability score are reported in Table 8. The influence of perceived stress did not reach statistical significance for any ability score.

**Table 8:** Standardized parameters of the latent change score structural equation models in Study 2.

	*g* LCS		S LCS		M LCS		C LCS	
	*β*	*p*	*β*	*p*	*β*	*p*	*β*	*p*
Intercept	1.775	.012	2.913	< .001	2.194	< .001	1.655	< .001
Perceived Stress	-.041	.432	.056	.359	-.046	.357	-.080	.141
	R LCS		V LCS		N LCS		F LCS	
	*β*	*p*	*β*	*p*	*β*	*p*	*β*	*p*
Intercept	2.208	< .001	3.199	< .001	2.329	< .001	1.702	.003
Perceived Stress	-.026	.593	-.037	.499	.034	.453	-.072	.288

*Note*. LCS = Latent change score. *g* = General Intelligence. S = Processing Speed. M = Memory. C = Creativity. R = Reasoning. F = Figural Ability. N = Numerical Ability. V = Verbal Ability.

## Discussion

Intelligence test results were lower in the pandemic 2020 sample than in the prepandemic 2002 and 2012 samples. The differences in test scores were large, with a difference in general intelligence of 7.62 IQ points between 2020 and 2002 (Analysis 1a). This difference did not appear to be a continuation of a longer decreasing trend. In contrast, we observed larger test scores in 2012 than in 2002 but lower scores in 2020. The difference between 2012 and 2020 was also substantial, with a difference in general intelligence of 6.54 points (Analysis 1b). The cross-sectional cohort comparisons therefore seem to corroborate previous results that regular schooling has a substantial impact on intelligence development and its absence is detrimental for intelligence test performance [9]. The difference in test scores was remarkably large. It may be the case that the student population was hit particularly hard by the pandemic, having to deal with both the disruption of regular schooling and other side effects of the pandemic, such as stress, anxiety, and social isolation [68]. Moreover, students are usually very accustomed to testing situations, which may be less the case after months of remote schooling.

Creativity scores were notably lower than other scores in 2002. It therefore seems like the nonsignificant difference in creativity between 2002 and 2020 was not due to creativity being unaffected by the pandemic, but instead due to creativity scores being low in 2002. This is supported by significantly higher creativity scores in 2012. Lower creativity in 2002 than in later years may be due to unfamiliarity with the testing format, changes in curricula, or changes in out of school activities.

Importantly, the overall results are inconsistent with one possible alternative explanation of decreasing intelligence test scores, namely, a reverse Flynn effect. Flynn observed a systematic increase in intelligence scores across generations in the 20^th^ century [69]. In some countries, a reversed Flynn effect with decreasing intelligence scores across generations has been observed in recent years [17, 70, 71]. This seems to be an especially plausible alternative explanation for the observed differences in test scores in our Analysis 1a. However, there are arguments against this alternative explanation. A reversal of the Flynn effect has not yet been observed in Germany. Instead, even in recent years, a regular positive Flynn effect has been reported [45, 72]. Moreover, a reverse Flynn effect is also inconsistent with our observation of increasing test scores from 2002 to 2012. We observed an increase in General Intelligence equivalent to .47 IQ points per year, which is slightly larger than the typically observed Flynn effect [73] or the Flynn effect observed in Germany [45]. The observed decrease in test scores from 2012 to 2020 with .82 IQ points per year for General Intelligence is also much larger than the reverse Flynn effect observed elsewhere (.32 IQ points) [74], making it unlikely that this effect alone could account for the observed decline.

The longitudinal results (Fig 9) showed an increase in test scores between the test (2020) and retest (2021). The magnitude of the increase is in line with the retest effects for intelligence testing that have been quantified meta-analytically (*d* = .33) [46]. In some cases the retest effects were larger than expected based on the meta-analysis (e.g., Processing Speed, Figural Ability). However, these cases were largely in line with a previous investigation of retest effects in a subsample of the BIS-HB standardization sample, [75] with no clear pattern of consistently larger or smaller retest effects in the present sample. These results indicate neither a remarkable decrease nor a “catching up” to previous cohorts.

Interestingly, we found no impact of perceived stress on the change in intelligence test scores. A possible explanation for the observed results is that stress levels were especially high in the first months of the pandemic, when there was the greatest uncertainty about the nature of the disease and lockdowns and school closures were novel experiences. Some evidence for a spike in stress levels at the beginning of the pandemic comes from tracking stress-related migraine attacks [76] and from a longitudinal survey of college students that was conducted in April and June 2020, finding the highest stress levels in April [77]. Moreover, teachers and students were both completely unprepared for school closures and online teaching at the beginning of the pandemic. The retest was conducted after a month-long period of regular schooling, followed by a now more predictable and better prepared switch to remote schooling that did not catch teachers and students off guard entirely. These factors may explain why intelligence performance did not drop further and why stress levels did not have an effect on the change in performance in the second test.

### Strengths and limitations

The present study has several strengths. To our knowledge, this is the first investigation of the development of intelligence test performance during the pandemic. Moreover, we used a relatively large, heterogeneous sample and a comprehensive, multidimensional intelligence test. We were able to compare the results of our sample with two highly similar prepandemic samples using propensity score matching. Last, we retested a large portion of the sample to longitudinally investigate the development of intelligence during the pandemic.

However, the present study also has several limitations that restrict the interpretation of the results. First, due to the pandemic affecting all students, we were not able to use a control group but had to rely on samples collected in previous years. Cohort effects cannot be completely excluded, although we tried to minimize their influence through propensity score matching and the use of two different prepandemic comparison groups. We could not control for potential differences in socioeconomic status (SES) between the samples because no equivalent measure was used in all three cohorts. It would have been beneficial to control for SES because of its influence on cognitive development and on the bidirectional relationship of intelligence and academic achievement [9]. SES differences between samples therefore may account for some of the observed test score differences. However, large differences in SES between the samples are unlikely because the 2012 and 2020 samples were drawn from the same four schools. Regarding the impact of SES on the longitudinal change during the pandemic in the 2020 sample, we did not have a comprehensive SES measure available. However, we had information on the highest level of education of parents. When adding this variable as a predictor in the LCA analyses, the results did not change, and parents’ education was no significant predictor of change.

Second, both measurement points of the study fell within the pandemic. A prepandemic measurement is not available for our 2020 sample. This limits the interpretation of the change in test scores over the course of the pandemic, even though we compared the observed retest effects with those found in meta-analysis and a previous retest-study of the BIS-HB.

Third, the 2020 measurement occurred only a few weeks after the summer break. It has often been shown that the summer break causes a decrease in math achievement test scores [78] as well as intelligence test scores [79]. However, this “summer slide” effect on intelligence seems to be very modest in size [80] and is therefore unlikely to be fully responsible for the large observed cohort differences in the present investigation.

Fourth, perceived stress was only measured by a short, retrospective scale. The resulting scores may not very accurately represent the actual stress levels of the students over the school year. Moreover, perceived stress was not measured at the first measurement point, so changes in stress levels during the pandemic could not be examined. This limits the interpretation of the absence of stress effects on changes in intelligence.

Fifth, the matched groups in Analysis 1b were somewhat unbalanced with regard to grade level (Table 1). The students in the 2020 sample tended to be in higher grades while being the same age. However, this pattern is unlikely to explain the differences in intelligence. The students in the 2020 sample tended to have experienced more schooling at the same age than the other samples, which would be expected to be beneficial for intelligence development [10, 11].

Sixth, there was some attrition between the first and second measurement of the 2020 sample. This was due to students changing schools or school classes, being sick or otherwise absent on the second day of testing or failing to provide parental consent for the second testing. It may be plausible that especially students with negative motivational or intellectual development changed school or avoided the second testing. This means that the improvement between the first and second time of measurement may be somewhat overestimated in the present analyses.

Seventh and last, only a modest percentage of the samples were matched in the PSM procedure because we followed a conservative recommendation for the caliper size [55] that yielded a very balanced matching solution. The limited common support somewhat diminishes the generalizability of the findings to the full samples.

### Implications

The pandemic and the associated countermeasures affected the academic development of an entire generation of students around the world, as evidenced by decreases in academic achievement [3]. Simulations predict a total learning loss between .3 and 1.1 school years, a loss valued at approximately $10 trillion [81]. Although we cannot make any causal claims with the present study, our results suggest that these problems might extend to students’ intelligence development. They point out that possible detrimental effects especially took place during the first months of the pandemic. Moreover, our longitudinal results do not point to any recovery effects.

As schooling has a positive impact on students’ cognitive development, educational institutions worldwide have a chance to compensate for such negative effects in the long term. As interventions aimed at the improvement of academic achievement also affect intelligence, [9] the decline in intelligence could be recovered if targeted efforts are made to compensate for the deficit in academic achievement that has occurred. Furthermore, schools could pay attention to offering intellectually challenging lessons or supplementary programs in the afternoons or during vacations, as intellectually more stimulating environments have a positive effect on intelligence development [82].

A second implication concerns current intelligence testing practice. If there is a general, substantial decrease in intelligence test performance, testing with prepandemic norms will lead to an underestimation of the percentile rank (and thus IQ) of the person being tested. This can have significant consequences. For example, some giftedness programs use IQ cutoffs to determine eligibility. Fewer students tested during (or after) the pandemic may meet such a criterion. If the lower test performance persists even after the pandemic, it may even be necessary to update intelligence test norms to account for this effect.

As discussed in the previous section, the present study has several limitations. The results can therefore only be regarded as a first indication that the pandemic is affecting intelligence test performance. There is a need for further research on this topic to corroborate the findings. It is obviously no longer possible to start a longitudinal project with prepandemic measurement points. However, the present article presented a way to investigate the effect of the pandemic if prepandemic comparison samples are available. Ideally, the prepandemic samples would have been assessed shortly before the pandemic onset to minimize differences between cohorts due to the (reverse) Flynn effect, changes in school curricula, or school policy changes. If a sample was assessed very recently before the pandemic, it may also be possible to retest the participants for the investigation of the pandemic effects. Although we cannot make any causal claims with the present study, our results suggest that COVID-19-related problems might extend to students’ cognitive abilities. As intelligence plays a central role in many areas of life, it would be important to further investigate differences between prepandemic and current student samples to account for these differences in test norms and for possible disadvantages by offering specific interventions.
